# Refinement and preliminary evaluation of two tablet‐based tests of real‐world visual function

**DOI:** 10.1111/opo.12658

**Published:** 2019-12-26

**Authors:** Pete R Jones, Iris Tigchelaar, Giorgia Demaria, Iain Wilson, Wei Bi, Deanna J Taylor, David P Crabb

**Affiliations:** ^1^ Division of Optometry and Visual Science School of Health Sciences, City University of London London UK; ^2^ Department of Ophthalmology University Medical Center Groningen University of Groningen Groningen the Netherlands; ^3^ Ocusweep Turku Finland; ^4^ Doctoral Program in Clinical Research University of Turku and Turku University Hospital Turku Finland; ^5^ Graduate School of Medical Sciences (Research School of Behavioral and Cognitive Neurosciences) University of Groningen Groningen the Netherlands; ^6^ Nuffield Laboratory of Ophthalmology Nuffield Department of Clinical Neurosciences University of Oxford Oxford UK; ^7^ Oxford Eye Hospital Oxford University Hospitals NHS Foundation Trust Oxford UK

**Keywords:** face discrimination, real world, tablets, visual function, visual search

## Abstract

**Purpose:**

To describe, refine, evaluate, and provide normative control data for two freely available tablet‐based tests of real‐world visual function, using a cohort of young, normally‐sighted adults.

**Methods:**

Fifty young (18–40 years), normally‐sighted adults completed tablet‐based assessments of (1) face discrimination and (2) visual search. Each test was performed twice, to assess test‐retest repeatability. *Post‐hoc* analyses were performed to determine the number of trials required to obtain stable estimates of performance. Distributions were fitted to the normative data to determine the 99% population‐boundary for normally sighted observers. Participants were also asked to rate their comprehension of each test.

**Results:**

Both tests provided stable estimates in around 20 trials (~1–4 min), with only a further reduction of 14%–17% in the 95% Coefficient of Repeatability (CoR_95_) when an additional 40 trials were included. When using only ~20 trials: median durations for the first run of each test were 191 s (*Faces*) and 51 s (*Search*); test‐retest CoR_95_ were 0.27 *d* (*Faces*) and 0.84 s (*Search*); and normative 99% population‐limits were 3.50 *d* (*Faces*) and 3.1 s (*Search*). No participants exhibited any difficulties completing either test (100% completion rate), and ratings of task‐understanding were high (*Faces*: 9.6 out of 10; *Search*: 9.7 out of 10).

**Conclusions:**

This preliminary assessment indicated that both tablet‐based tests are able to provide simple, quick, and easy‐to‐administer measures of real‐world visual function in normally‐sighted young adults. Further work is required to assess their accuracy and utility in older people and individuals with visual impairment. Potential applications are discussed, including their use in clinic waiting rooms, and as an objective complement to Patient Reported Outcome Measures (PROMs).

## 1 Introduction

Traditional clinical measures of basic visual function, such as visual acuity (VA), do not necessarily reflect the patient's experience, or the impact of vision loss on patients' lives.^1^ As a result, patient‐reported outcome measures (PROMs) are often used as secondary outcome measures (and sometimes as primary outcome measures) in ophthalmic clinical trials.^2^, ^3^, ^4^, ^5^ However, discrepancies have been found between self‐reports and actual performance on real world tasks.^6^, ^7^, ^8^ Moreover, perceived problems with visual function might be influenced by other factors, such as an individual's lifestyle or personality.^9^, ^10^ Methods for directly assessing performance on everyday visually‐guided tasks may be more appropriate for assessing the impact of vision loss. Furthermore there is substantial clinical interest in developing more ‘patient friendly’ tests that can quickly and easily assess real‐world visual function.^11^, ^12^, ^13^, ^14^, ^15^

Our previous work suggests that face discrimination and visual search are two tasks that are particularly important to patients, and are often impaired in people with glaucoma^16^, ^17^, ^18^, ^19^, ^20^, ^21^ or age‐related macular degeneration.^22^, ^23^, ^24^ Currently, however, the equipment required for these tests is bulky, and the tests themselves often relatively time‐consuming, making them appropriate only for use in research. Rapid, tablet‐based versions of these real‐world measures would be more clinically applicable. Tablet tests are particularly attractive as they are inexpensive, and because patients in the waiting room can potentially complete them while they wait to be seen – thereby minimising any burden to patients and staff. Indeed, the idea of using tablet‐based activities to more productively utilise the time patients spend in waiting areas is a growing area in other medical disciplines. For example, tablets are being increasingly used in health‐care to collect  questionnaire/PROM data,^25^, ^26^ as an educational tool,^27^, ^28^, ^29^, ^30^ or for functional testing.^31^ However, this concept remains relatively unexplored in ophthalmology.

In this paper, we describe two rapid, tablet‐based tests for assessing face discrimination and visual search, both of which we have made freely available online (see *Methods*). In the present work, we piloted these tests on a large number of young, normally‐sighted ‘control’ participants. This allowed us to refine the tests, assess usability, and to establish a normative database prior to conducting further studies with patient populations. The objectives of this study were to: (1) measure completion rates, test‐retest reliability, test‐durations, and ease‐of‐use for two, novel, tablet‐based tests of visual function; (2) refine the tests on the basis of these measurements, and; (3) establish a normative database of expected scores for a young, normally‐sighted population.

## 2 Methods

### 2.1 Overview

Normally‐sighted young adults performed two tablet tests designed to assess real‐world visual function. One test (*Faces*) measured participants' ability to discriminate between four human faces (‘spot the odd one out’). The other test (*Search*) measured participants' ability to locate a particular object in a crowded scene (‘find the matching object’). Each test was performed twice, in order to assess within‐visit (intrasession) test‐retest repeatability. Note that within‐visit variability is more likely to reflect the inherent fluctuations due to measurement error (‘intrinsic noise’), whereas between‐visit variability may also include true vision fluctuations, and so may be greater. The order of the tests was interleaved, ABAB, with the starting test determined randomly for each participant. Each test was set to run for a fixed — relatively large — number of trials, allowing us to determine, *post hoc*, how many trials were actually required in order to obtain a statistically stable measure of performance. After testing, participants were also asked to rate the ease and clarity of each test, while before testing measures of basic vision (acuity, contrast sensitivity) and cognition (digit recall) were also taken, for comparison.

### 2.2 Participants

Participants were 50 adults aged 18–40 years (32 female; mean [standard deviation] age: 25 [5] years) with normal or correct‐to‐normal vision. All participants completed both tests twice. However, for the *Faces* test, data from only 30 individuals are reported. The other 20 participants performed variants of the *Faces* test (e.g., different adaptive algorithm parameters) that were found to substantially less efficient than the final version reported here.

Normal vision was defined as no history of eye disease, and (1) binocular best‐corrected letter acuity ≤0.2 *log*MAR (tested with an Early Treatment Diabetic Retinopathy Study [ETDRS] chart); (2) binocular best‐corrected Pelli‐Robson contrast sensitivity ≥1.5 *log*CS (tested with Pelli‐Robson chart); and (3) a passing score on the 38‐plate Ishihara pseudoisochromatic test (Handaya, Tokyo, Japan, 2011 edition). An additional five individuals were recruited (total *N* = 55), but were excluded from the study as they failed to meet all three criteria.

All screening was conducted in a well‐lit room by authors IT and GD. Letter acuity and contrast sensitivity charts were presented in a standard lightbox, were scored letter‐by‐letter,^32^ and terminated after more than 50% incorrect responses on a single row. Measures of contrast sensitivity and colour vision loss were included since both tablet tests involved real‐world stimuli containing a range of spatial frequencies and contrast levels, and since the *Search* task further included significant chromaticity cues. For completeness, it would also have been desirable to screen for near visual acuity. Its absence as an inclusion criterion in the present study is unlikely to have been problematic given the present cohort of young adults, but reduced near visual acuity may be a substantial confounding factor for older users.

Participants were recruited via advertisements placed around City, University of London. The study was approved by the Ethics Committee for the School of Health Sciences, City, University of London (#ETH1819‐0532), and was carried out in accordance with the Declaration of Helsinki. Written informed consent was obtained from all participants prior to testing, and participants were given £15 compensation for their time.

### 2.3 Equipment

**Figure 1:**
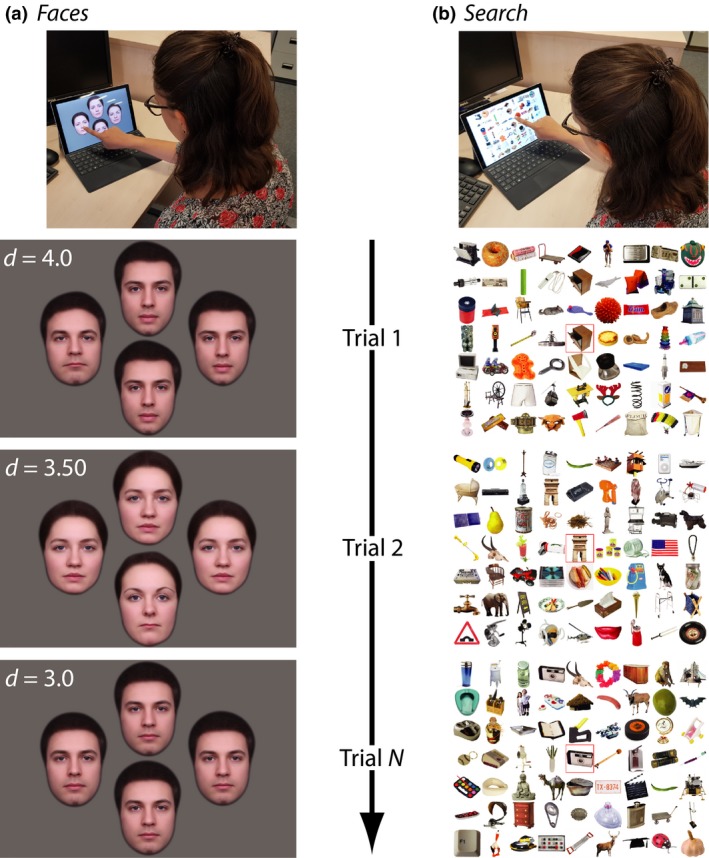
Methods. Setup and example trials for the (a) *Faces* test, and (b)* Search* test. The ‘*d*’ value for the Faces test indicate the magnitude of dissimilarity between the target face and the standard faces, with lower values implying more difficult discriminations (see body text for details). In the *Search* test the cue always appeared centrally, within a red box, while the location of the matching target was varied systematically between trials, in random order.

Both tests were run on a Microsoft Surface Pro 4 (http://www.microsoft.com): a touchscreen tablet computer with an IPS (in‐plane switching) screen measuring 26 cm × 17.3 cm (*Figure* 1). Participants were positioned approximately 50 cm from the screen at the start of each test (using a tape measure); however, viewing distance was not strictly constrained. On both tests, the screen was viewed binocularly, and participants were allowed to move their eyes freely. Participants responded by touching the tablet screen, as detailed separately for each test, below.

### 2.4 Task 1: Faces

On each trial, the participant was asked to identify (touch) the ‘odd one out’, from a set of four faces (four‐alternative forced‐choice discrimination; 4AFC). Three of the faces were identical within a given trial (the Standard), though varied randomly between trials. The fourth face (the Target) varied from the Standard by parametrically manipulating photographs of real faces. The source of our face photographs was a dataset from the University of Stirling [Stirling ESRC 3D Face Database: (http://pics.stir.ac.uk)] containing 50 frontal images faces [25 male], each manually labelled using Psychomorph software (http://pics.psych.stir.ac.uk/ESRC/software.html) 33 with 127 feature points (points on the face that define head shape, hairline and internal feature locations). These 127 features could then be varied by a percentage to create progressively more dissimilar versions of a particular reference image. Faces were masked at presentation to display only the face and removing the ears (which were often observed to exhibit image artefacts after warping). For further technical details regarding the generation and manipulation of the face stimuli see previous works by Logan and colleagues,^34^ after which the present work was based.

The Standard face and the location of the Target varied randomly between trials (see *Figure* 1 *a*). There was no time limit, but participants were encouraged to respond “as quickly and accurately as you can”. Depending on whether the participant answered correctly or incorrectly on the previous trial, the degree of similarity between the faces was decreased or increased after every trial, using a QUEST adaptive algorithm.^35^ The test ran for a fixed number of 50 trials. However, it was anticipated that fewer trials would be sufficient to obtain a stable estimate of performance (see *Results*).

The outcome measure was *threshold*: the smallest difference between the faces that the participant could detect reliably. This was defined mathematically as the Maximum Likelihood Estimate (MLE) of the mean of the posterior density function, which was computed after every trial. As illustrated in *Figure* 1 *a*, threshold values typically ranged between 3–4, with bigger numbers indicating poorer performance (lower sensitivity). Note that Threshold was measured in units of dissimilarity, *d*,  which express the Euclidean distance between the Standard and the just‐noticeably‐different Target face.

### 2.5 Task 2: Search

On each trial, a random image (the Reference) was presented in the centre of the screen, and remained visible throughout the trial. After 1 s, 62 additional images then appeared also on the screen, spaced uniformly on a 7 × 9 grid (see *Figure* 1 *b*). One of these 62 images (the Target) was identical to the central Reference image. Participants were asked to locate the matching Target image as quickly as possible, and to touch it. All 63 images (including the Reference image) varied on every trial, drawn randomly from a previously described database of 2400 real world objects^36^ (see *Figure* 1 *b* for examples).

Unlike with the *Faces* test, the difficulty of the task did not vary trial‐by‐trial. However, the location of the Target was systematically manipulated: appearing once at each possible grid location over the course of 62 trials. In principle, participants could therefore use a process of elimination from previous trials to inform where the Target was most likely to appear next. In practice, however, there was no evidence that participants attempted to do so, or were even aware of this possibility. Moreover, the order of target locations was randomised each time the test was run.

Every possible location was tested once (62 trials). However, it was anticipated/hypothesised that a subset of test locations might be sufficient (see *Results*). The outcome measure was ***Response Time*** (RT), in seconds. This was measured once at each test location, and the overall median RT was computed as a summary measure of performance.

This test represents a modified version of a previous test that we developed to explore the effects of non‐neovascular (dry) age‐related macular degeneration (AMD).^37^ It was modified in the present work to ensure that the Reference image remained visible in the centre of the screen throughout, in order to minimise the memory component of the test. We have in the past also used cluttered, real‐world scenes to assess object search performance^18^, ^23^ (e.g., using 2D photographs or 3D virtual environments). A uniform grid of discrete objects was employed in the present test, however, as it allowed the target location to be systematically varied between trials, and would allow us to more easily manipulate the stimuli in future (e.g., to refine the stimulus set, or in order to examine a particular domain of object categories).

### 2.6 Measure of cognition

Participants also completed the “Digit Span” subtest from the Wechsler Adult Intelligence Scale IV (WAIS‐IV) test battery. The Digit Span test measures participants' ability to repeat increasing sequences of numbers forwards and backwards.^38^ Low scores may indicate problems with working memory (or a general lack of motivation^39^).

### 2.7 Analysis

Standard inferential statistical analyses were performed using MathWorks MATLAB R2016b (https://www.mathworks.com/products/matlab). When reporting key statistics, bootstrapping was used to compute 95% Confidence Intervals (CI_95_; *N* = 20 000; bias‐corrected and accelerated method).

### 2.8 Availability of test materials and study data

Study data for both tests are available as Supplemental Material. This dataset also includes the measurements of vision (Acuity, Contrast Sensitivity), cognition, and usability, for each participant.

The *Face* test was programmed in Python (http://www.python.org) by author IW, using the OpenSesame toolbox^40^ (http://www.osdoc.cogsci.nl). The source code is freely available online for non‐commercial use at: https://www.bitbucket.org/iainrwilson/facediscrimination.

The *Search* test was programmed in C# by author WB, and an executable is freely available online for non‐commercial use at: https://github.com/CrabbLab/CrazySearch.

## 3 Results

### 3.1 Test refinement

**Figure 2:**
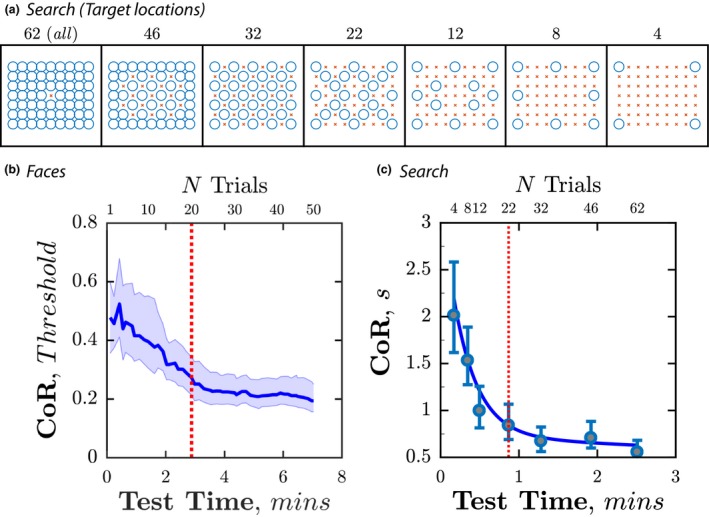
Test refinement. Within‐subject (test‐retest) measurement variability, as a function of *N* trials for the (b) *Faces* test and (c) Search Test. Coefficients of Repeatability were derived using Bland‐Altman analysis, as detailed in Figure 5, below. Panel (a) shows the target locations (blue circles) associated with each *Search* grid.

Each test was intentionally run for longer than piloting indicated was necessary. To determine how many trials were actually required to obtain stable estimates of performance, we examined how test‐retest variability (the 95% Coefficient of Repeatability; CoR) varied as a function of test duration/number of trials (*Figure* 2). For *Faces*, this involved simply analysing the first *N* trials (i.e., since the adaptive algorithm provides an updated, maximum‐likelihood estimate of discrimination ability after every trial). For *Search*, data were analysed from progressively more sparse subsets of spatially‐distributed locations, as shown in *Figure* 2 *a*.

Unsurprisingly, increasing the number of trials resulted in greater measurement precision (*Figure* 2 *b‐c*). However, precision improved rapidly for the first 20 (*Faces*) or 22 trials (*Search*), and more gradually thereafter. For example, in the *Faces* task CoR_95_ decreased by 43% as the number of trials increased from *N* = 1 to *N* = 20, with only a further 17% reduction by *N* = 50. Similarly, in the *Search* task CoR_95_ decreased by 58% from *N* = 4 to *N* = 22, with a further 14% reduction by *N* = 62.

The ideal test duration will depend on the level of precision required. However, we anticipate that ~20 trials will be sufficient for most clinical purposes. This corresponds to approximately 1–3 min (see below). Tests of a longer duration would also likely be unacceptable to people waiting in routine clinics. We therefore report data only for these subsets of ~20 trials in the remainder of the manuscript.

### 3.2 Normative values

**Figure 3:**
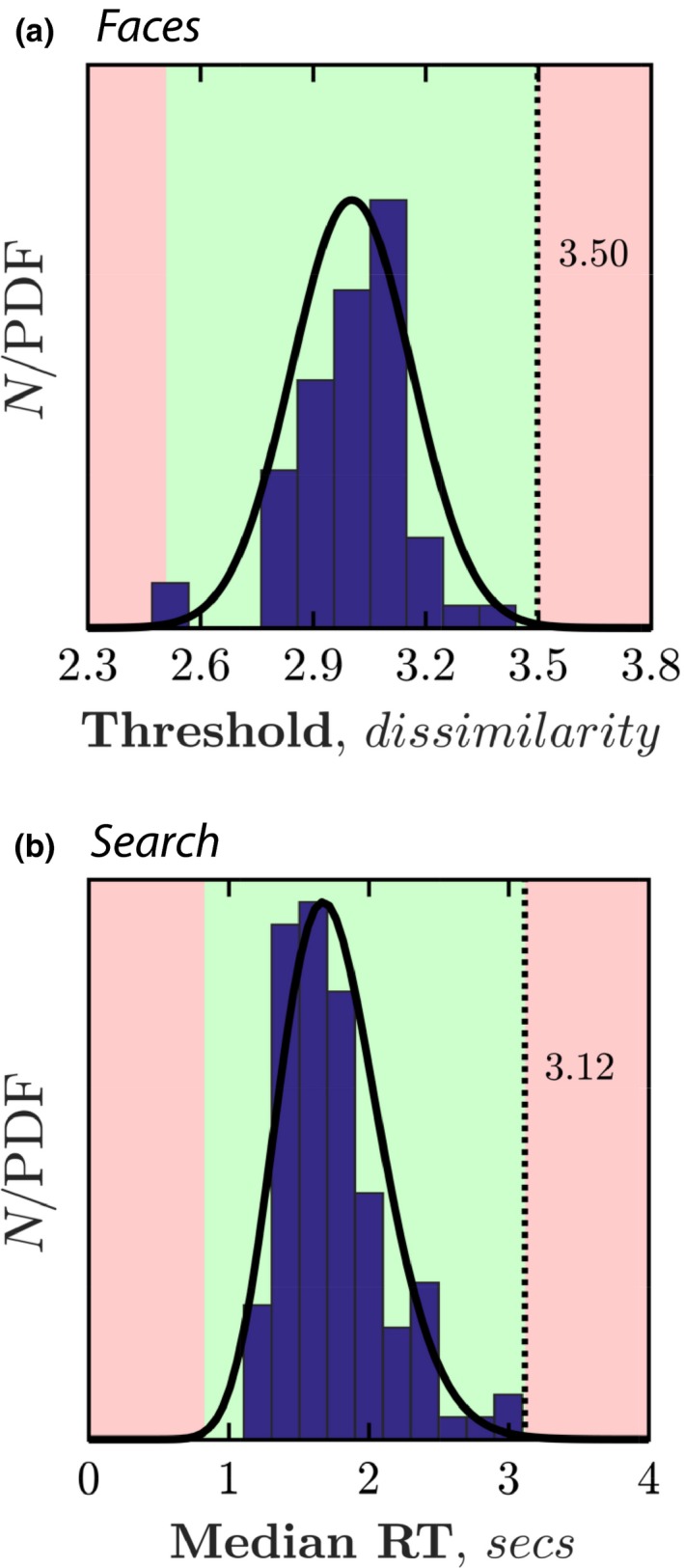
Normative data for the (a) *Faces* test, and (b) *Search* test. Curves show maximum likelihood fits of appropriate probability density functions (Faces: Gaussian PDF. Search: Gamma PDF). Dashed vertical lines indicate the cutoff point, below which 99% of normally‐sighted participants would be 5 expected to score.

There was no systematic difference in performance between the first and second run, either for *Faces* (*p* = 0.11) or *Search* (*p* = 0.42). Accordingly, data from both runs were concatenated to produce the normative distributions shown in *Figure* 3 *.* Appropriate probability distributions (black lines) were fitted to the raw data. These were used to determine the 99% upper‐bound point (dashed vertical line): the cut‐off point below which 99% of young, visually‐normal participants would be expected to score. These values were 3.50 (*Faces*) and 3.1 s (*Search*). Values greater than this may indicate abnormal test performance.

**Figure 4:**
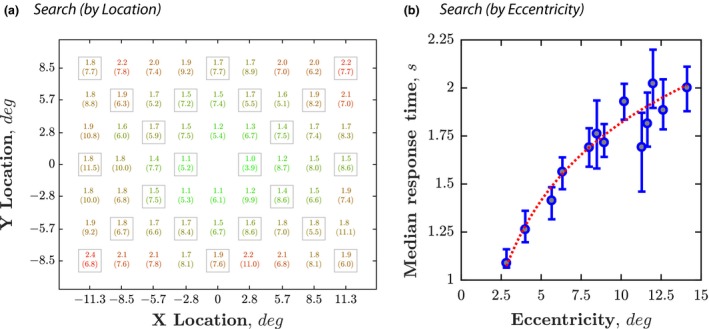
Pointwise normative data for the Search task, as a function of (a) Screen location; (b) Eccentricity from the centre. Grey boxes indicate the subset of 22 points that were used in all other figures and analyses. Data for the other 40 locations also given for completeness. Eccentricities computed assuming a viewing distance of 50 cm. Red dashed line indicates the best fitting power function [*y* = −13.14*x* ^−0.05^ + 13.56; Adjusted *R* ^2^ = 0.90]. Individual markers indicate median response times with 95% confidence intervals.

For *Search* (only) it is also possible to consider performance for individual spatial locations. This could be important if, for example, attempting to detect localised visual field loss. Accordingly, *Figure* 4 shows normative median values, and the 99% upper cut‐off value for each location (computed in the same way as for the overall median RT, in *Figure* 3 *b*). As highlighted in *Figure* 4 *b*, there was a clear effect of eccentricity, with participants being slower to locate more peripheral targets.

### 3.3 Test‐retest reliability

**Figure 5:**
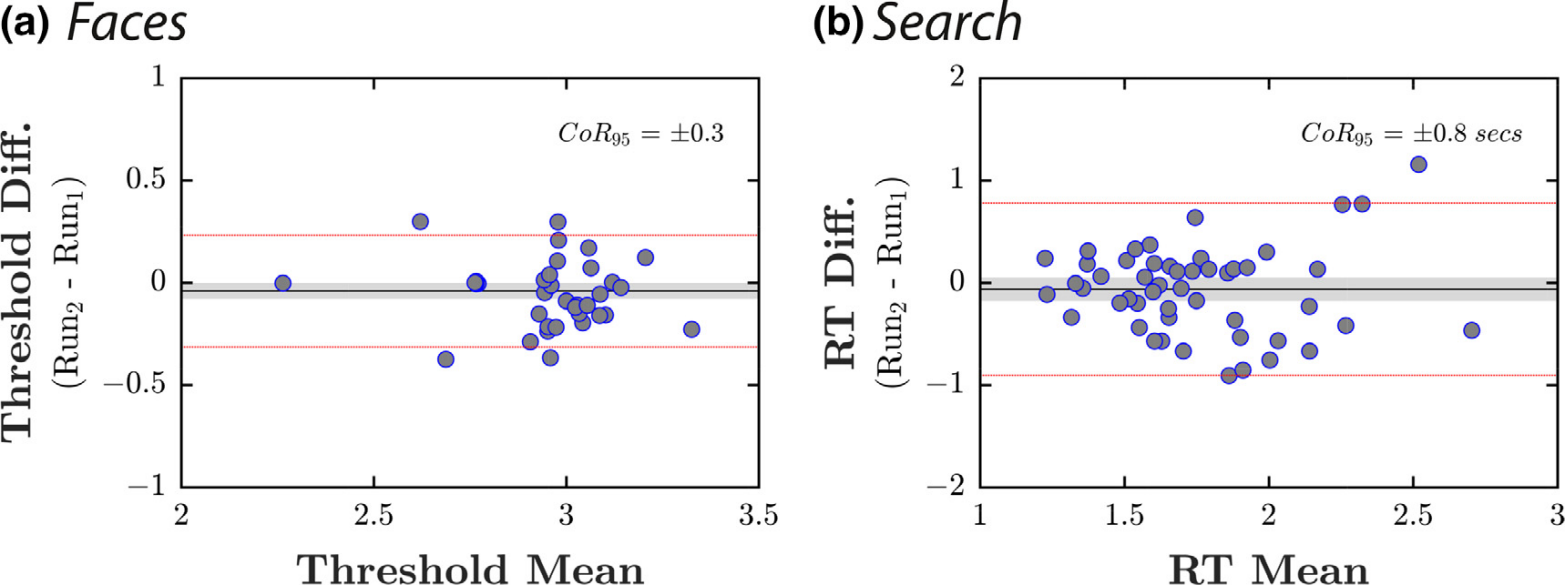
Bland‐Altman analyses of retest repeatability for (a) Faces and (b) Search. Each marker represents a single participant. Grey shaded regions show 95% confidence intervals around the mean. Dashed red lines indicate the 95% limits of agreement. Text (top‐right) gives the 95% Coefficient of Repeatability (CoR_95_).

As shown in *Figure* 5, the Coefficient of Repeatability {± CI 95%} was 0.27 {0.22, 0.35} for *Faces*, and 0.84 {0.71, 1.07} for *Search*. As is evident by inspection, there was no systematic effects of learning or fatigue. Measurement error tended to be approximately normally distributed, although on the *Search* task there was a tendency for variability to increase as a function of overall reaction time.

### 3.4 Test duration

For *Faces*, median {CI_95%_} test duration was 191 {168, 228} s for the first run, and 155 {139, 186} for the second: a statistically significant difference (*t‐*test: *p* = 0.042) of 19%. For *Search*, median {CI_95%_} test duration was 51 {46, 56} s for the first run, and 47 {45, 50} s for the second: a non‐significant difference (*t‐*test: *p* = 0.16).

### 3.5 Usability & completion rate

All participants (100%) completed both tests twice, with no participants exhibiting/reporting any difficulties. Participants were asked to rate how clearly they understood what to do on each test, on a scale from 0 (incomprehensible) to 10 (very understandable). Ratings of comprehension were remarkably high for both tests with a mean {CI_95%_} rating of 9.6 {9.1, 9.8} for *Faces*, and 9.7 {9.2, 9.8} for *Search*.

### 3.6 Relationships with cognition and basic vision

**Figure 6:**
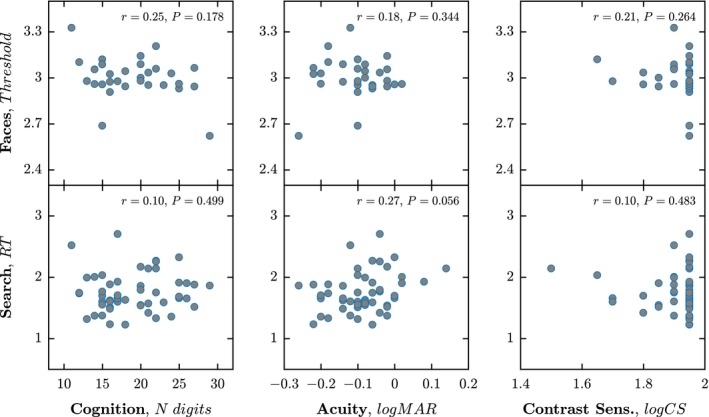
Scatter plots showing the relationships with cognition (Digital Recall) and basic vision (Acuity, Contrast Sensitivity). Each marker indicates a single participant, with scores across the two runs of each test mean‐averaged. Text (top‐right) gives the results of independent Spearman's rank correlations.

As shown in *Figure* 6, there was no significant association between performance on either test and with: (1) Digit Span general cognition; (2) logMar letter acuity; or Pelli‐Robson contrast sensitivity (see *Figure* 6 for *p* values) – although there was a trend towards an association between acuity and performance on the *Search* task (*p* = 0.056; *r* = 0.27). In those 30 participants who performed both tests, there was also no correlation between scores on the *Faces* and *Search* task (*r* = 0.18; *p* = 0.16).

## 4 Discussion

The purpose of the present study was to describe, refine, evaluate (in normally sighted young adults), and provide normative control data for two tablet‐based tests of real‐world visual function, both of which we have made freely available online. The results established that both tests were capable of providing stable estimates of visual function in around 20 trials (~1–2 min). They also defined cutoff points for what constitutes ‘normal’ performance, and showed that the tests could be performed easily by young adults, who, anecdotally, often regarded them as ‘games’ (in contrast to the vision screening ‘tests’ that preceded them). This last point is crucial, as tests must be must be simple, intuitive, and engaging if to be completed autonomously by patients, without the need for costly technicians.^41^

There was no relationship between performance on the tablet tests, and scores on a test of cognitive function (*Digit Span*). This is encouraging, as it suggests that they are measuring genuine perceptual abilities, rather than general cognitive ability or effort. However, this result should be taken with caution, since no participants scored outside of normal limits on the cognitive test, and all behavioural measures are inevitably susceptible, to some degree, to cognitive factors such as motivation, understanding, and compliance. Likewise, there was little or no association with more basic tests of vision, suggesting that these novel measures may be able to provide additional information over and above what can be inferred from standard measures of acuity or contrast sensitivity. Again though, this may in part reflect the highly homogenous nature of the cohort; in the limiting case it is, for example, necessary to have some basic level of acuity to be able to even attempt to perform either test. Ultimately, additional data from patients are required to establish the clinical utility of these measures, over and above more basic measures of visual function (see *Limitations*, below).

### 4.1 Potential applications

The new tablet‐based tests described here are simple and relatively enjoyable, do not require a trained operator to supervise, and can be run using inexpensive commercial equipment that is easy to maintain or replace. They would therefore be ideal for giving to patients in waiting rooms, where many individuals would likely welcome the distraction, and where the tests can effectively provide ‘free’ data – without extending appointment durations or further burdening clinical staff. For example, we are currently examining whether they can be used in diabetic macular edema [DME] clinics to identify individuals experiencing real‐world difficulties despite mild VA loss.^42^ We are also exploring their use with age‐related macular degeneration (AMD) patients to assess the real‐world impact of progressive central vision loss. Ultimately, however, we envisage their potential applications to be multifarious. As such, we have made the tests freely available online for people to use and develop (see *Methods*).

### 4.2 Comparison to previous literature

The present work is timely, as a number of other tablet‐based tests have recently been reported, designed to measure various more basic aspects of visual function, such as visual acuity,^15^, ^41^, ^43^, ^44^ contrast sensitivity,^45^, ^46^, ^47^, ^48^, ^49^ visual fields,^12^, ^50^, ^51^, ^52^, ^53^, ^54^, ^55^, ^56^ stereopsis,^57^ and colour vision.^58^, ^59^ What distinguishes the tests described in the present study is that they are intended to measure ‘high‐level’ function on everyday tasks. They can therefore be thought of as a complementary, more objective analog to traditional PROMs, and so may be particularly well suited to flagging up those patients who are experiencing everyday difficulties not captured by more basic measures of visual function, such as acuity or contrast sensitivity.^42^, ^60^

On a practical level, the novel measures described in the present work are also much easier to administer than many of these more basic measures, since we anticipate there will be no need to calibrate the luminance or chromaticity or display (i.e., unlike contrast sensitivity), or to precisely control the viewing distance or ambient lighting of the observer (i.e., unlike acuity). Though it is perhaps interesting to note that both low‐ and high‐level functional tests could be potentially be performed with the same tablet devices. For example, we describe elsewhere a tablet‐perimeter (Eyecatcher) that uses the exact same tablet computer as described in the present work.^56^ Elsewhere, the use of tablets as a means of sharing clinical data between a patient's eyecare team has also been explored.^61^

With respect to traditional PROMs, it is worth stressing that the present tests are not intended as a like‐for‐like replacement. Asking patients directly remains the best way to ascertain how someone feels about their condition, and PROMs, when used well,^62^ have been shown capable of providing important insights into the everyday difficulties that patients face.^63^, ^64^ PROMs are, however, potentially limited by individual differences in personality, knowledge and expectations of disease,^65^ as well as by differences in lifestyle (e.g., with some individuals not reporting difficulties with a particular task domain because they now avoid it altogether, or have develop adaptation strategies to cope with their condition). We might therefore learn something complementary by assessing actual performance in surrogates of tasks that patients would encounter every day.^66^

### 4.3 Limitations & future work

The primary limitation of the present study is that we only assessed young people with healthy vison. These data allowed us to refine the measures, perform a preliminary assessment of feasibility, and provide limits on what constitutes normal performance. In future, however, it will be necessary to collect data from patients to more fully assess their speed, reliability, and relationship to basic measures of visual function. Doing so may also suggest further refinements. For example, the *Faces* test, with a median test duration of around 3 min may already be ‘at the limit’ of what is clinically practicable, and it may be necessary to further shorten the test, either by reducing the number of trials, and/or by integrating a Bayesian prior into the adaptive algorithm.^67^

A related limitation is that we only examined young adults. Older adults are often slower and less accurate at locating objects in cluttered scenes.^68^, ^69^, ^70^ In future, it would therefore be helpful to collect additional normative data for older adults performing the *Search* task. There may also be age‐related changes in performance on the *Faces* test also. Although interestingly older adults, while sometimes exhibiting difficulties recalling faces,^71^ often appear no worse than younger adults at discriminating between simultaneously presented faces, after correcting for differences in vision.^72^

A final limitation is that testing in the present work took place in a controlled, university environment. This is potentially quite different to a busy clinic. In future, it will be necessary to conduct more extensive studies to assess the feasibility — and clinical utility — of deploying such tests in a practicing clinic. These are questions which we currently investigating, and intend to report data from in due course.

## Conflict of interest

The authors report the following professional relationships, and no conflicts of interest: PR Jones, None; I Tigchelaar, None; G Demaria, None; I Wilson, None; W Bi, None; DJ Taylor, None; DP Crabb, Roche, Allergan (F), Allergan, Santen, THEA, Bayer(R).

## Supporting information


**Data S1.** Raw data for each participant, including primary outcome measures for the faces and search task, as well as measurements of vision (Visual Acuity, Contrast Sensitivity), cognition, and usability.
